# Decreased complexity of glucose dynamics in diabetes in rhesus monkeys

**DOI:** 10.1038/s41598-018-36776-4

**Published:** 2019-02-05

**Authors:** Richard Raubertas, Jeremy Beech, Wendy Watson, Steven Fox, Scott Tiesma, David B. Gilberto, Ashleigh Bone, Patricia A. Rebbeck, Liza T. Gantert, Stacey Conarello, Walter Knapp, Tasha Gray, Larry Handt, Cai Li

**Affiliations:** 1 Early Development Statistics, Merck & Co., Inc., Kenilworth, NJ 07033 USA; 2 Laboratory Animal Resources, Merck & Co., Inc., Kenilworth, NJ 07033 USA; 3 Pharmacology, Merck & Co., Inc., Kenilworth, NJ 07033 USA; 4 Data Science International, 119 14th St NW, St. Paul, MN 55112 USA; 5 Translational Imaging Biomarkers, Merck & Co., Inc., Kenilworth, NJ 07033 USA

## Abstract

Until recently, preclinical and clinical work on diabetes has focused on the understanding of blood glucose elevation and its detrimental metabolic sequelae. The advent of continuous glucose monitoring (CGM) technology now allows real time monitoring of blood glucose levels as a time series, and thus the exploration of glucose dynamics at short time scales. Previous work has shown decreases in the complexity of glucose dynamics, as measured by multiscale entropy (MSE) analysis, in diabetes in humans, mice, and rats. Analyses for non-human primates (NHP) have not been reported, nor is it known if anti-diabetes compounds affect complexity of glucose dynamics. We instrumented four healthy and six diabetic rhesus monkeys with CGM probes in the carotid artery and collected glucose values at a frequency of one data point per second for the duration of the sensors’ life span. Sensors lasted between 45 and 78 days. Five of the diabetic rhesus monkeys were also administered the anti-diabetic drug liraglutide daily beginning at day 39 of the CGM monitoring period. Glucose levels fluctuated during the day in both healthy and diabetic rhesus monkeys, peaking between 12 noon – 6 pm. MSE analysis showed reduced complexity of glucose dynamics in diabetic monkeys compared to healthy animals. Although liraglutide decreased glucose levels, it did not restore complexity in diabetic monkeys consistently. Complexity varied by time of day, more strongly for healthy animals than for diabetic animals. And by dividing the monitoring period into 3-day or 1-week subperiods, we were able to estimate within-animal variability of MSE curves. Our data reveal that decreased complexity of glucose dynamics is a conserved feature of diabetes from rodents to NHPs to man.

## Introduction

The global diabetes epidemic continues to impose substantial health and financial burdens on society. According to the most recent estimate by The International Diabetes Federation^1^, 1 in 11 adults suffers from diabetes worldwide, and 12% of global health expenditure is spent on diabetes, reaching $727 billion. In the US, care for people with diagnosed diabetes accounts for 1 in 4 health care dollars, and more than half of that expenditure is directly attributable to diabetes^2^.

The hallmark of diabetes is elevated blood glucose. Preclinical and clinical research on diabetes has focused on understanding the mechanisms of blood glucose elevation as well as its detrimental metabolic sequelae. All therapies developed since the discovery of insulin about a century ago have focused on decreasing blood glucose levels.

The development of continuous glucose monitoring (CGM) technology, introduced to the market about 20 years ago^3,4^, has provided richer information about fluctuations in blood glucose on relatively short time scales. Clinical benefits include real-time display of glucose levels and rate of change of glucose, alerts for actual or impending hypo- and hyperglycemia, around the clock coverage, and the ability to characterize glycemic variability^5^.

CGM data also allows more fundamental research on the dynamics of blood glucose on short time scales. Recent work has applied multiscale entropy (MSE) analysis, a data analysis technique for quantifying the complexity of a time series^6^, to CGM data from diabetic humans and animals. MSE analysis was introduced to overcome the limitations of traditional approaches to measuring the complexity of biological signals. One example is the analysis of fluctuations of human heartbeat intervals under physiologic and pathologic conditions. MSE analysis consistently indicates a loss of complexity of heart beat with aging, with a cardiac arrhythmia (atrial fibrillation) and a life-threatening syndrome (congestive heart failure) each exhibiting a distinct MSE curve profile. These results led to the hypothesis of a general “complexity-loss” theory of aging and disease^6,7^. Indeed, recent work published in 2014^2,3^ demonstrated a reduction in complexity of glucose dynamics in diabetics compared to healthy subjects. Since then, decreases in the complexity of glucose dynamics have been demonstrated in diabetic mice and rats^8^ as well as in additional clinical studies^9–11^. Although CGM studies have been conducted in non-human primates^12,13^, complexity of glucose dynamics was not analyzed. In addition, it is not known if diabetes medications that reduce glucose levels also correct the decreased complexity of glucose dynamics.

In this report, we describe results of performing CGM for 6–11 weeks in both healthy and diabetic rhesus monkeys. Consistent with the above studies in other species, we show that diabetic monkeys have lower complexity of glucose dynamics than healthy monkeys at MSE time scales up to 30 minutes. We also extend these results in several ways. At longer time scales the difference in complexity between diabetic and healthy animals diminishes. We show that complexity of glucose dynamics varies by time of day, more strongly for healthy animals than for diabetic animals. We examined the effect of the anti-diabetic drug liraglutide on complexity in diabetic animals, and found that 2–4 weeks of treatment did not consistently change complexity. And we studied the within-animal variability of MSE curves by analyzing 3-day and 1-week subperiods of the data.

## Results

### Overview of CGM data

Figurementary Figure S1 shows the timeline of CGM measurements and procedures for the four healthy and six diabetic monkeys. Supplementary Table S1 summarizes the CGM data available for each animal after preprocessing. The length of usable glucose data ranged from 45 to 78 days. The measurement frequency was one glucose value per second.

Most analyses reported here are based on the first five weeks of monitoring data, since liraglutide treatment in the diabetic animals began during the sixth week. Supplementary Figure S2 shows the mean and standard deviation of glucose values over the first five weeks of monitoring for each animal. Both means and standard deviations were generally much higher for diabetic animals compared to healthy animals. Diabetic animal 161283 had intermediate values and did not cluster with either the healthy animals or the other diabetic animals.

### Circadian changes of glucose levels in both healthy and diabetic rhesus monkeys

**Figure 1:**
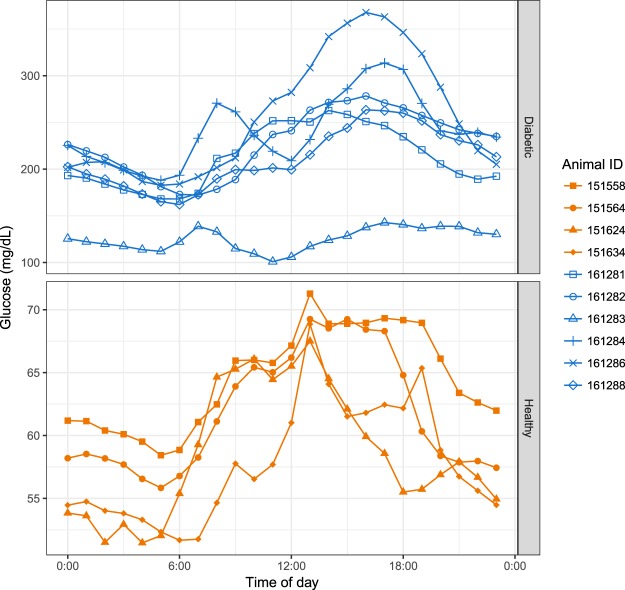
Mean glucose levels by time of day in healthy and diabetic rhesus monkeys. Days were divided into one-hour periods. For each animal, mean glucose was calculated for each one-hour period. Upper and lower panels denote diabetic (“Diabetic”, n = 6) and healthy (“Healthy”, n = 4) rhesus monkeys profiled.

Figure 1 shows mean glucose levels for each animal by time of day. Circadian changes of glucose levels were evident, with glucose levels lowest between midnight and 6 am, increasing during the day, peaking in the afternoon and decreasing in the evening. Although this was true for all the animals as a group, glucose profiles of individual animals did vary. For example, diabetic animals 161283 and 161284 had a second peak between 6 am and 9 am. The pattern of circadian glucose changes was similar in diabetic and healthy animals, although the morning increase was generally steeper and the peak earlier for the healthy animals.

### Complexity of glucose dynamics is decreased in diabetic rhesus compared to healthy rhesus

**Figure 2:**
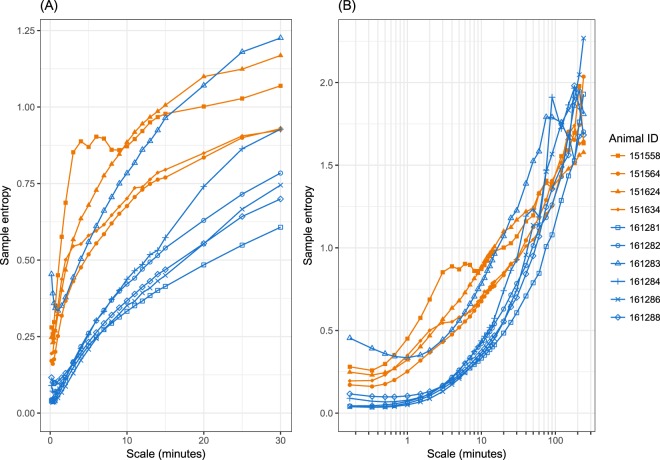
MSE curves for glucose for individual animals, using the first five weeks of CGM data. **(A)** Scale factors up to 30 minutes; **(B)** Scale factors up to 4 hours (log scale).

Figure 2 shows MSE curves for glucose for the individual healthy and diabetic animals. With the exception of animal 161283, curves for diabetic animals were consistently below those for healthy animals at time scales up to 30 minutes. Beyond 30 minutes the difference between groups diminished and disappeared at scales greater than one hour. In addition, the shapes of the curves differed between groups. Curves for healthy animals tended to rise more rapidly at short time scales and less rapidly at longer time scales than curves for diabetic animals. Animal 161283 was unique in that the shape of its MSE curve was similar to that of the other diabetic animals, but the height of the curve was more similar to that of healthy animals. This animal was also unique in its glucose levels and variability (Supplementary Fig. S2).

Supplementary Figure S3A and Table S2 summarize the MSE curves in each group by their means and standard errors. Differences in means were statistically significant (p < 0.05) for time scales between 40 seconds and 20 minutes. If animal 161283 was excluded from the diabetic group, the differences in means became larger and were statistically significant at all time scales from 10 seconds to 30 minutes. Similarly, mean MSE-AUC_10s-30m_ was smaller for diabetic animals than for healthy animals (Supplementary Fig. S3B and Table S3).

### Time of day effects on MSE

**Figure 3:**
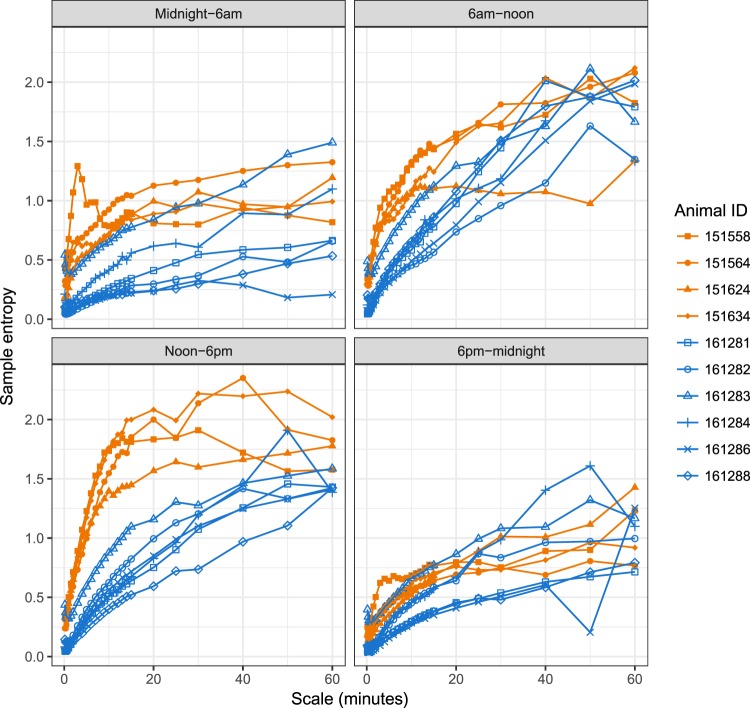
MSE analysis of glucose by time of day, using the first five weeks of CGM data. Days were divided into four 6-hour periods and MSE analysis was carried out separately for each period for each animal.

Days were divided into four 6-hour periods, starting at midnight. Glucose MSE and MSE-AUC_10s-30m_ were calculated using just data from each of those periods, aggregated across the first five weeks of monitoring. Figure 3 shows MSE curves for each animal by time of day. Overall, healthy animals had larger MSE than diabetic animals in each period. However the size of the difference varied, being smallest between 6 pm and midnight and largest between noon and 6 pm. In addition, the heights of the curves varied by time of day, especially for the healthy animals. This can be seen clearly in Supplementary Fig. S4 and Table S4, which summarize MSE curves by their MSE-AUC_10s-30m_. For the healthy animals MSE-AUC_10s-30m_ varied strongly during the day, with a peak during the noon-6 pm period. For the diabetic animals MSE-AUC_10s-30m_ was lower and less variable across periods, with a peak between 6 am and noon. In this respect animal 161283 was more similar to the other diabetic animals than to the healthy animals.

### Within- and between-animal variability of MSE

The first five weeks of monitoring data for each animal were divided into consecutive 3-day or 1-week periods, and MSE analysis was carried out separately within each period. Supplementary Fig. S5 shows how estimated MSE curves varied from period to period for each animal. This represents the intrinsic within-animal variation in MSE over time, in the absence of treatment interventions. Curves did vary noticeably in height, but curve shape for a given animal was more stable. Using MSE-AUC_10s-30m_ to summarize each MSE curve, Supplementary Fig. S6 shows how this varied across periods, and Figure S7 quantifies the variation by its standard deviation (SD). The period-to-period SD was smaller for 1-week periods than for 3-day periods for eight of 10 animals. This is as one would expect, since a longer monitoring period should lead to more stable estimates of MSE.

Mixed model analysis was used to obtain overall estimates of within- and between-animal SD’s of MSE-AUC_10s-30m_ for each period length, pooling across animals. The SD’s for the between-animal component of variance were 5.9 and 5.1 for 3-day and 1-week periods respectively. The SD’s for the corresponding within-animal variances were 4.8 and 3.7. These values can be used to estimate the gain or loss in information from longer or shorter monitoring periods, which is discussed below.

### Glucose lowering effect of liraglutide in diabetic rhesus

As a proof of concept to detect glucose lowering by CGM in response to an antidiabetic compound, liraglutide was administered to five of the diabetic rhesus monkeys via daily subcutaneous injection, beginning on CGM day 39 and continuing for 28 days (Figure S1B). The sixth diabetic animal, 161281, was not treated because it was already receiving insulin. Pharmacokinetic data for liraglutide in the rhesus suggested that daily injection at 15 µg/kg should have a C_max_ of 13.2 nM (49.5 ng/mL) and a T_max_ of 6.7 hours (Supplementary Fig. S8). In the clinic, maximum concentrations of liraglutide following subcutaneous administration are achieved at 8–12 hours post dosing, with mean peak (C_max_) exposure at 35 ng/mL for a subcutaneous single dose of 0.6 mg, increasing proportionally over the therapeutic dose range of 0.6 mg to 1.8 mg^14,15^.

**Figure 4:**
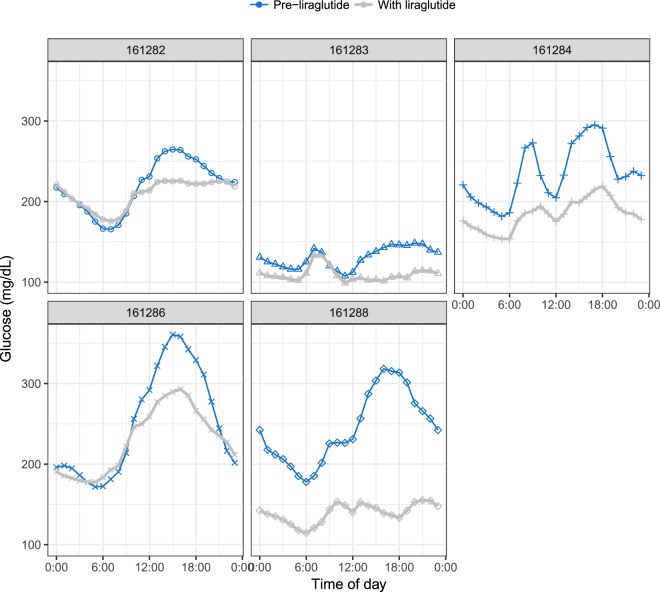
Mean glucose levels by time of day for five diabetic rhesus monkeys treated with liraglutide. Days were divided into one-hour periods. For each animal, mean glucose was calculated for each one-hour period, and the means at a given time of day were then averaged across all days during liraglutide treatment (gray lines) or an equal number of days preceding treatment (blue lines, see also Supplementary Table S1).

The overlap of liraglutide treatment with the usable CGM data period varied from 16 to 28 days (Supplementary Table S1). We compared this liraglutide treatment period with the immediately preceding period of the same length. Table 1 summarizes the data for each animal over the full treatment and pre-treatment periods. Mean glucose declined for all animals, by 12–102 mg/dL (5–42%). Standard deviation also decreased; there was no consistent change in coefficient of variation (standard deviation as a proportion of the mean). Figure 4 shows mean glucose levels by time of day, before and during liraglutide treatment. Liraglutide reduced the afternoon peak in glucose in all five animals; it reduced glucose at all times of day in two animals (NHP161284 and 161288).

**Table 1:** Glucose levels in diabetic animals before and during liraglutide treatment.

Animal ID	Pre−liraglutide			With liraglutide			Change in mean	
	Mean	SD	CV	Mean	SD	CV		%
161282	220.5	50.6	0.23	208.2	46.3	0.22	−12.2	−5.5
161283	131.2	31.9	0.24	109.6	27	0.25	−21.6	−16.5
161284	234.2	61.3	0.26	183.1	56.3	0.31	−51.1	−21.8
161286	256.4	88	0.34	236.6	59.6	0.25	−19.9	−7.7
161288	243	68.1	0.28	140.9	33.7	0.24	−102.1	−42.0

*Pre−liraglutide* period immediately precedes the start of the liraglutide treatment period and is of the same length as the *With liraglutide* period (see also Supplementary Table S1).

SD = standard deviation; CV = coefficient of variation (SD/mean). Means and SD’s are in mg/dL.

### Effect of liraglutide on MSE

**Figure 5:**
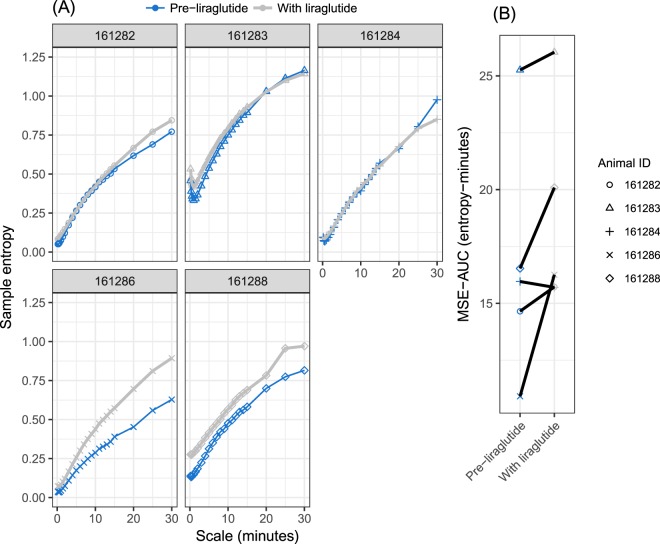
MSE and MSE−AUC before and during liraglutide treatment of diabetic animals. The pre-liraglutide period immediately precedes the liraglutide treatment period and is of the same length (see also Supplementary Table S1). **(A)** MSE curves; **(B)** MSE - AUC_10s−30m._

Figure 5 shows MSE curves and MSE-AUC_10s-30m_ for the liraglutide treatment and pre-treatment periods. For three animals there was essentially no difference in MSE between the two periods. For animals 161286 and 161288 the MSE curve was higher during the treatment period, and for the latter the treatment period curve approached the level of the healthy animal curves in Fig. 2A. However in both cases the curve shape remained more similar to the diabetic animal curves in Fig. 2A. Note that animal 161288 had the largest decrease in mean glucose during treatment (Table 1, Fig. 4), but the change in glucose levels for 161286 was the second smallest.

## Discussion

This study carried out high-frequency (1 value/second), extended duration (6–11 weeks) monitoring of blood glucose in healthy and diabetic rhesus monkeys. Using multiscale entropy analysis to assess the complexity of glucose dynamics, we found substantially lower complexity in five diabetic animals compared to four healthy animals, at time scales up to at least 30 minutes. This is consistent with previous results in rodents^8^ and humans^16,17^ and thus extends those results to a nonhuman primate. We also observed that the shapes of the MSE curves for these diabetic animals differed from those of the healthy animals, being flatter at small time scales and steeper at larger time scales. An intriguing observation was that the MSE curve for a sixth diabetic animal had an overall height similar to the healthy animals but a shape more similar to the other diabetic animals; this animal also had blood glucose levels intermediate between the healthy and other diabetic animals.

From these results and data from our earlier work in monogenic models of diabetes in mice and rats^8^, it appears that there are both glucose level-dependent and glucose level-independent sources affecting complexity of glucose dynamics in diabetes. The glucose level-independent component is best illustrated by studies in diabetic ZDF rats, where the decrease in complexity occurred prior to any appreciable rise of glucose levels relative to normal rats^8^. The glucose level-dependent component is illustrated by the further decrease in complexity of glucose dynamics in diabetic ZDF rats when transitioning to frank diabetes^8^, and by the observation in the current NHP study of an animal with glucose levels (~125 mg/dL) intermediate between healthy (~65 mg/dl) and overtly diabetic (>200 mg/dL) that also exhibited an intermediate profile in complexity of glucose dynamics (Fig. 2). Glucose level-dependence is further supported by analyses of complexity during intra-day periods, where maximum differences in complexity of glucose dynamics coincided with largest differences in blood glucose levels (Figs 3 and S4). Clinical data also demonstrated that type 1 diabetics exhibit the lowest complexity of glucose dynamics, consistent with the contribution of both genetic factors and high glucose levels toward observed decrease in complexity of glucose dynamics^10^.

The relatively long monitoring period in this study allowed us to extend MSE analyses beyond earlier work in rodents and humans in several ways. First, it allowed us to examine MSE time scales up to four hours, rather than the 30 minutes reported in earlier work. We observed that the difference between healthy and diabetic animals diminished beyond 30 minutes and the MSE curves for the two groups were indistinguishable by time scales of about one hour (Supplementary Figure S3). It is not clear what this implies about the physiology of glucose control. But as a pragmatic matter of study design, it suggests that focusing on time scales no larger than 30 minutes will maximize sensitivity for detecting differences in the complexity of glucose dynamics when studying diabetic rhesus monkeys.

Second, we were able to split the data by time of day and look for circadian patterns in MSE. In healthy animals we observed strong variation in entropy levels by time of day, with lowest complexity between 6 pm and midnight, and rising to a peak between noon and 6 pm. In diabetic animals the variation by time of day was smaller and slightly different in pattern, with highest complexity between 6 am and noon. The net result was that there was minimum difference from healthy animals between 6 pm and midnight, and maximum difference between noon and 6 pm. This may reflect greater strain on the mechanisms of glucose control during times when animals are eating, which the healthy animals were able to cope with but the diabetic animals were not.

Third, the length of monitoring provided an opportunity to explore the effect of an approved anti-diabetic compound on MSE. A question of great interest in diabetes drug development is whether complexity is a useful supplement to traditional measures such as glucose level or HbA1c as a marker of the effectiveness of treatment: Do drugs that reduce glucose levels also shift complexity toward levels observed in healthy individuals? Does normalizing complexity provide any clinical benefit above and beyond the benefit of normalizing glucose levels? As an informal exploration of the first question, we administered 15 μg/kg of the GLP-1 agonist liraglutide daily to five of the diabetic monkeys, beginning on day 39 of monitoring. Liraglutide treatment reduced mean glucose level in all five animals. However there was no change in MSE curves for three of the animals, and only one of the other two had a substantial upward change. Although this was obviously not a definitive test of the effect of liraglutide on complexity, the data do suggest that any effect is not dramatic, or requires more than a few weeks of treatment to appear. Because liraglutide treatment extended beyond the life span of the glucose sensor for most animals, it was not possible to determine if longer treatment would allow a more unequivocal conclusion about its effect on complexity. Our data also raise the question of whether a treatment regimen that emphasized glucose lowering in the afternoon would be of particular value with respect to both peak hyperglycemia and glucose dynamics. A comparison of liraglutide with other anti-diabetes drugs (such as sulphonyl urea) might shed light on this question.

Finally, we examined the within-animal variability of MSE; that is, how much natural variation there is in an animal’s MSE curve over the course of five weeks. We considered curves estimated using 3 days or 1 week of monitoring data. There was substantial variability in the levels of the curves from period to period, although the shape of the curve for a given animal seemed to be less variable. We used a mixed model analysis to quantify the within- and between-animal variability of the summary measure MSE-AUC_10s-30m_ for 3-day and 1-week monitoring periods. The estimated within/between standard deviations were 4.8/5.9 for 3-day periods and 3.7/5.1 for 1-week periods. As expected, a longer monitoring period led to less variable MSE-AUC_10s-30m_ values, and variability within animals was less than between animals.

These SD estimates are based on only 10 animals and so not very precise, but they can be used to illustrate the potential information gain associated with longer monitoring times. For example, if one were planning a study to compare MSE-AUC_10s-30m_ between two groups of animals (as in the above comparison of healthy and diabetic animals), the relevant variability is the total variance: the sum of the squares of the between- and within-animal SD’s. The required sample size to detect a specified difference in group means with a given statistical power is proportional to this total variance. Estimated total variances were 56.9, 39.2, and 22.8 for 3-day, 1-week, and 5-week periods. Since our analysis included only one 5-week period per animal, we cannot estimate separate within- and between-animal variances, but can still estimate the total variance. These imply that extending the length of monitoring from 3 days to 1 week would reduce the number of animals required by a factor of 39.2/56.9 = 0.69, and by a further factor of 22.8/39.2 = 0.58 by extending it from 1 week to 5 weeks.

For a crossover study, in which each animal is tested under both of the conditions to be compared, the relevant variability is the within-animal variance. In that case, extending the length of monitoring from 3 days to 1 week per condition would reduce the required number of animals by a factor of (3.7^2^/4.8^2^) = 0.59.

It is worth noting that although the CGM data from healthy and diabetic NHPs was only used to determine the complexity of glucose dynamics by MSE analysis in this manuscript, the same dataset could also be used to quantify glucose variability (GV), a metric focusing on the amplitude and frequency/timing of glucose variations^18,19^. For example, principal component analyses of a pool of twenty-five well-established indices for GV from CGM time series of both type 1 and type 2 diabetes patients revealed that up to 10 different GV indices were sufficient to preserve more than the 60% of the variance originally explained by all 25 indices^20,21^. However, a correlation between GV and complexity of glucose dynamics might not exist, as two signals can have the same degree of statistical variability (i.e., the same global variance and coefficient of variation), but very different complexity properties (https://physionet.org/tutorials/cv/).

CGM is still a largely underpenetrated market, with physicians reporting only ~28% of Type 1 patients and ~5% of Type 2 patients on CGM as of 2017. Robust adoption in the Type 2 patient population is expected, in particular with the launch of Dexcom’s 10-day wear, non-adjunctive, no-fingerstick, real-time, G6 integrated continuous glucose sensor, iCGM, in the US late in 2Q18^22,23^. The availability of tools to directly analyze data from various CGM devices manufactured by Dexcom, Abbott, and Medtronic should provide ample opportunities to interrogate CGM data on changes in complexity of glucose dynamics when patients are treated with various pharmacological treatments. This may ultimately shed light on best treatment options for the more than 400 million diabetes patients waiting for a cure.

## Materials and Methods

### Animals and animal handling

Light cycles were from 6 am–6 pm. NHPs were fed a Purina LabDiet 5037, manufactured by Purina (Saint Louis, MO). Merck Institutional Animal Care and Use Committee approved all the experiments and that all experiments were performed in accordance with relevant guidelines and regulations. Sex, age, and weight of the animals at the time of surgery are listed in Table S1.

### Liraglutide formulation and dosing

Liraglutide was obtained from Novo Nordisk at a concentration of 6 mg/mL; it was diluted into 1 mg/mL stock solution, then diluted further 1:10 to 100 µg/mL solution as working stock. Vehicle used was 1 mM Sodium Phosphate (pH = 8) and 5% Mannitol. To determine the pharmacokinetics (PK) of liraglutide in rhesus, liraglutide was injected into (N = 3) rhesus monkeys subcutaneously at a dose of 0.2 mg/kg. Blood samples were obtained at 0.08, 0.25, 0.50, 1.00, 2.00, 4.00, 6.00, 7.00, and 24.00 hours post-dose. Liraglutide levels were measured using a cAMP cell based bioassay. The assay’s lower limit of quantification was ~1 nM thus preventing accurate PK determination at doses lower than 0.2 mg/kg. However based on the strong dose proportionality seen in GLP-1 peptide analogues, the expected level of liraglutide at a 15 µg/kg sc dose in the rhesus was 13.2 nM at C_max_. Dosing solutions were quantitated to be at 97.3% of the nominal concentrations. Five of the six diabetic monkeys received liraglutide; animal 161281 did not because it was already insulin deficient and on insulin injection to maintain glucose. Compound was given daily subcutaneously at 15 µg/kg in the morning each day (~8–9 am). Lira dosing started on Day 39 of the CGM period and continued for 28 days.

### DSI device and device-related procedures

Surgery procedure: Rhesus monkeys underwent surgery for placement of the DSI HD-XG glucose sensor into the right carotid artery. Animals were initially sedated with 10 mg/kg ketamine IM, intubated and anesthesia was maintained through isoflurane inhalation. All monkeys received 0.01 mg/kg Buprenorphine IM, 4.4 mg/kg carprofen SC and 20 mg/kg ceftiofur SC preoperatively. A 3–4 centimeter skin incision was made on the right, mid-lateral neck over the carotid artery where the pulse was palpated. A 1–2 centimeter segment of the right carotid artery was isolated and a tight ligature was placed distally towards the head around the exposed section of vessel. A second ligature was pre-placed, but not tightened, proximally towards the chest around the exposed section of vessel to prevent excessive bleeding when the vessel was cut. A small incision was made in the artery between these two ligatures. The sensor was inserted into the lumen of the vessel and advanced towards the heart approximately 8–9 centimeters. The pre-placed ligature was tightened to prevent bleeding around the sensor. A third ligature was secured around the sensor between the previously placed ligatures and further secured around the vessel to prevent the sensor from migrating. The telemetry body was secured to the underlying musculature in the right lateral neck near the sensor entry into the artery. Monkeys received 2 mg/kg bupivacaine locally at the skin incision. All incisions were then closed routinely. The animals were removed from the isoflurane anesthesia to allow for recovery and were monitored until they were sternal and stable. Animals received 4.4 mg/kg carprofen PO or IM once the day following surgery and were observed daily for the next 10 days to assure a normal recovery. A reference electrode and telemetry body was placed in the subcutaneous tissue near the catheter entry point as recommended by DSI.

The implanted sensor transmits its signal to a repeater located on the same animal on the collar. That repeater then immediately retransmits the HD-XG data on a different frequency to a repeater receiver up to 8 meters away. Each repeater and repeater receiver used in the study had a unique transmission frequency such that telemetry interference would be avoided (and cages adjacent to each other could be supported). Glucose sensor values were reported once per second.

All repeater receivers were connected to one or more Data Exchange Matrix (DEM) which in turn were connected to a computer running Dataquest A.R.T. version 4.36 software. This software enabled both acquisition of the raw data and calibration of the glucose signal to collected reference data. Each repeater required a battery change at intervals of no more than 5 days.

The conversion of the raw glucose signal from nanoamps into the desired glucose units of mg/dL was performed by an algorithm that used two types of calibration references, and time-based interpolation based upon these references.

Multipoint and single point calibrations were performed to obtain reference blood glucose values. Multipoint calibrations require both high and low glucose reference values to be collected such that a linear regression can be performed to determine slope and intercept coefficients. For multipoint calibrations, ivGTTs were performed on Day 0 and Day 48 for healthy NHPs and on Day 0, 34, and 66 for diabetic NHPs via ivGTT. NHPs were anesthetized with ketamine before 1 mL/kg of 50% dextrose was infused intravenously over a 2-minute period. Glucose levels were obtained with a glucometer at baseline, and at approximately 2, 5, 10, and 15 minutes after dextrose infusion. Single point calibrations require an individual reference value of blood glucose so that it can be compared to the existing calibration and if needed sensitivity loss can be corrected. Single point calibrations were performed every 5 days or less.

Visual examination of the raw glucose signal for declines in level or dynamic range was used to estimate when the glucose sensor had reached the end of its service life. This defined the end of the usable CGM data for that animal.

### Multiscale entropy (MSE) analysis

Details about the definition and calculation of MSE are given in the Supplementary Materials and Methods. MSE-AUC_10s-30m_ is the area under an MSE curve between scale factors of 10 seconds and 30 minutes, and was used as a single-number summary of the curve.

#### Comparison of healthy and diabetic animals

Comparisons between healthy and diabetic animals were made using data from the first five weeks of monitoring, because treatment of diabetic animals with liraglutide began during the sixth week. MSE curves were calculated for each animal. The curves were summarized for each group by the average and standard deviation of sample entropy at each scale factor. Group means at each scale factor were compared using two-sided t-tests with the Welch-Satterthwaite adjustment to degrees of freedom. The latter avoids the assumption of equal variability in the two groups. MSE-AUC_10s-30m_ was also calculated for each animal, and group means were again compared using a t-test. No multiplicity adjustment was applied to p-values.

#### Effect of liraglutide on glucose and MSE

Five diabetic animals were treated with liraglutide for four weeks, beginning during the sixth week of monitoring. For four animals the end of usable glucose monitoring data came before the end of liraglutide treatment, so the length of the liraglutide treatment period in the data varied from 16 to 28 days. For each animal the period of the same length immediately preceding the start of liraglutide was used as the comparator (‘pre-liraglutide’) period. For each animal MSE curves and MSE-AUC_10s-30m_ were calculated for the pre-liraglutide and liraglutide periods and compared visually.

#### Time of day effects

Days were divided into four 6-hour periods, starting at midnight. Mean glucose, glucose MSE, and MSE-AUC_10s-30m_ were calculated using just data from each of those periods, aggregated across the first five weeks of monitoring. (That is, when calculating MSE for the midnight-6am period for example, data at other times of day were essentially treated as missing.) Only one-fourth as much data was available for each time of day period as for the full time series, so the maximum scale factor for MSE was reduced to 1 hour. Results were summarized for healthy and diabetic animals and the groups were compared visually and with t-tests as in section 4a above.

#### Within- and between-animal variability of MSE

The first five weeks of monitoring data for each animal were divided into consecutive 3-day or 1-week periods, and MSE analysis was carried out separately within each period. The maximum scale factor was set to 1 hour for 1-week periods and 30 minutes for 3-day periods. The standard deviation of MSE-AUC_10s-30m_ across these periods was calculated to describe the natural variability of MSE within a given animal over time. In addition, mixed effects models were used to produce pooled estimates of within- and between-animal variability of MSE-AUC_10s-30m_. The model for each period length included group (healthy or diabetic) as a fixed effect, and a random effect for animals. The standard deviation for the random effect estimated the between-animal component of variability and the residual standard deviation estimated within-animal variability.

## Electronic supplementary material

Supplementary Information
