# Supplementary material for: Decreased complexity of glucose dynamics in diabetes in rhesus monkeys
Source: Sci Rep. 2019 Feb 5;9:1438. doi: 10.1038/s41598-018-36776-4 (PMC6363759; doi:10.1038/s41598-018-36776-4)
Supplement: Supplementary file 1 — Supplementary Information [file 41598_2018_36776_MOESM1_ESM.pdf]

Decreased complexity of glucose dynamics in diabetes in rhesus monkeys

Richard Raubertas[1,*], Jeremy Beech[2], Wendy Watson[2], Steven Fox[3], Scott Tiesma[4], David B. Gilberto[2], Ashleigh Bone[2], Patricia A. Rebbeck[2], Liza T. Gantert[5], Stacey Conarello[3], Walter Knapp[2], Tasha Gray[2], Larry Handt[2,*], Cai Li[3,*]

[1]Early Development Statistics, [2]Laboratory Animal Resources, [3]Pharmacology, [5]Translational Imaging Biomarkers, Merck & Co., Inc., Kenilworth, NJ 07033, USA

[4]Data Science International, 119 14th St NW, St. Paul, MN 55112 USA

*Correspondence:

Richard Raubertas (richard_raubertas@merck.com)
Larry Handt (larry_handt@merck.com)
Cai Li (cai.li@merck.com)

Supplementary Materials and Methods

1. MSE analysis

a. Overview of MSE

Multiscale entropy analysis, introduced by Costa et al[1], is built upon a quantity called sample entropy[2], which we describe first. The sample entropy of a regularly spaced time series of length *N* is defined using two parameters: a subsequence length, *m*, and a similarity threshold, *r*. Consider any two subsequences of length *m* of the time series. (The subsequences may overlap, as long as they do not start at the same position.) The subsequences are said to match if the *i*-th element of one subsequence differs from the *i*-th element of the other by no more than *r*, for all $i = 1, \ldots, m$. Let *B* denote the number of matching pairs of subsequences when we consider all possible pairs of length-*m* subsequences from the first *N*-1 observations of the series. Next, repeat the process of counting matches, but use subsequences of length *m*+1instead of *m*, and consider all possible such subsequences taken from the full series of *N* observations. Let *A* denote the number of matching pairs among them. Then the sample entropy is the negative natural logarithm of the ratio A/B. See Richman and Moorman [2] for details and explicit formulas. Intuitively, sample entropy is the negative log of a conditional probability: given that two subsequences are similar in their first *m* elements, what is the probability that they are also similar at element *m*+1?

Multiscale entropy calculates this sample entropy after applying various amounts of averaging to the original time series. The averaging is determined by a set of scale factors, which may be specified either as numbers of observations or amounts of time. For each scale factor, the time series is partitioned into non-overlapping segments of that length, and each segment is summarized by its average. This is referred to as *coarse-graining* the series. The result is a new time series of averages, for which sample entropy is calculated. MSE analysis consists of examining how sample entropy varies across a set of scale factors.

b. Choice of MSE parameters

Application of MSE analysis requires choosing values for the parameters *m* and *r*, and the set of scale factors to use. We followed Costa et al [3] and Chen et al [4] and used *m*=2 and *r* equal to 0.15 times the standard deviation of the time series. The smallest possible scale factor is set by the time resolution of the series being analyzed, in our case 1 second. However we considered it unlikely that biologically interesting fluctuations in glucose occur at such short time scales, so we took the lower end of scale factors to be 10 seconds. At the upper end, scale factors are limited by the length of the series: coarse-graining a series with a scale factor of *t* converts a series of length *N* to one with length *N/t*, and when *t* is large the resulting series may not be long enough for reliable estimation of sample entropy. Since we had weeks of monitoring data available, we were able to consider a range of scale factors up to 4 hours: 10, 20, 30, 40, 60, 90 seconds; 2-15, 20, 25, 30, 40, 50, 60, 75, 90 minutes; 2, 2.5, 3, 3.5, and 4 hours.

c. Complexity index and MSE-AUC

MSE analyses are usually presented as a graph of sample entropy against scale factor, with points connected to form a curve. To summarize the curve into a single number, Costa et al [3] proposed the complexity index, the sum of sample entropy values over a set of equally spaced scale factors. We generalized this idea by using the area under the MSE curve (MSE-AUC) over a specified range of scale factors. This accommodates unequally spaced scale factors. Costa et al [3] used scale factors up to 30 minutes, so we focused on AUC from 10 seconds to 30 minutes, denoted $\text{MSE-AUC}_{10s\text{-}30m}$. Areas were calculated using the trapezoidal rule.

d. Missing values

Missing values in the data series had to be dealt with at both the coarse-graining and sample entropy steps. For coarse-graining the question is how many missing values to allow in a segment of the time series before calling the average for that segment missing. Setting this to 0 means that even a single missing value in, say, 60 seconds worth of data will cause the average for that segment of data to be considered missing. The segment therefore will not contribute to calculation of sample entropy at that time scale. In our 1-second glucose data there were many gaps of a few seconds or less scattered throughout the series. Choosing a small allowable missing fraction introduced many missing values into the coarse-grained series, drastically reducing the effective sample size for the sample entropy step. At the other extreme, setting the allowable missing fraction to 100% means that even if only 1 second worth of data is available in a 60 second segment (for example), that one value will be used to represent the segment. This seems inconsistent with the rationale for a multiscale analysis, which is supposed to examine the effect of averaging across multiple data points. We therefore chose an intermediate value of 40% as the maximum allowed missing fraction.

When the coarse-grained series contains missing values, they need to be allowed for in the calculation of sample entropy. The approach we applied was that if any subsequence of length m+1contained a missing value, then it was not included when counting matches for subsequences of length m+1, and its first m elements were not included when counting matches for length m (counts A and B in section 1a above). This ensured that every pair of subsequences included in B was also potentially included in A, maintaining the conditional probability interpretation of A/B.

e. Software

Software for calculation of MSE was derived from the publically available C program by M. Costa at https://www.physionet.org/physiotools/mse/mse.c. We modified the program to handle missing values as described above, to avoid integer overflow when analyzing long data series, and to integrate it into the R statistical programing system (R version 3.4.1, R Core Team, 2017).

2. Data preprocessing

a. End of usable data

The glucose sensor has a limited life span. During the course of technology development at DSI, the most common causes of sensitivity loss were found to be 1) degradation of enzyme function, and/or 2) development of a sheath over the sensor tip. Enzyme function degradation is accelerated by high glucose levels, attributed to excess H_2O_2 generation that causes decay in the activity of glucose oxidase. Less common reasons for signal loss include degradation or aggressive fibrinous growth over the reference electrode. For each animal the graph of raw sensor output was examined to determine declines in current or dynamic range indicating loss of sensitivity, and a cutoff date for usable glucose data was chosen[5]. We note that for one of the healthy animals, ID 151624, the sensor signal dropped and became flatter from approximately day 29 to day 40 of monitoring, then recovered. This may have been due to accumulation of biomass over the sensor. However because the cause of the anomaly was uncertain, we chose to include that data in our analyses (through day 35, as for the other healthy animals).

b. Missing values

Missing (unrecorded) glucose sensor values occurred for all animals throughout the monitoring period. In some cases gaps in measurement were due to the animal being out of range of the receiver for manual glucose calibrations. In other cases the reason for a gap was unknown. The proportion of missing values ranged from a few percent for animals 151564 and 161281 to over one-third for animal 161284. Most of the missing values occurred as frequent short gaps in the data that were widely

dispersed throughout the monitoring period: the median gap length was only 1 to 3 seconds across animals, and the median length of runs of non-missing observations between gaps ranged from 4 to 81 seconds. The default preprocessing by the device manufacturer reports only averages over 10-second intervals so most of these gaps are not visible; we chose to deal with them explicitly as describe above.

In addition, some observations were intentionally masked (set to missing) for the following reasons. (a) To reduce possible artifacts in glucose dynamics associated with animal handling procedures, we masked observations from 5 minutes before to 15 minutes after a glucometer reading, and from 5 minutes before to 45 minutes after ketamine administration or an ivGTT. (b) Glucose values at time points when there was no body temperature reading were masked. (c) Single-point (1-second) spikes in reported glucose were masked as being biologically implausible. A spike was defined as a large change in value (up or down) immediately followed by a large change in the opposite direction. The thresholds for a spike were a difference of ±10 mg/dL or greater from the preceding and following non-missing observations, provided that also represented a relative change of ±30% or greater. Percent changes were calculated relative to a 5-observation centered running median of the data values. (d) Reported glucose values less than 10 mg/dL were masked as biologically implausible.

Supplementary Figures

Supplementary Fig. S1. CGM timelines for four healthy rhesus monkeys (S1A) and six diabetic monkeys (S1B). Sedation/battery change/glucometer reading days are marked with the red arrows pointing up; glucometer readings by cage side on non-sedated monkeys are marked with black arrows pointing up. Two (healthy NHPs) or three (diabetic NHPs) ivGTTs were performed for the purpose of multi-point calibration of the CGM glucose sensor and indicated by arrows pointing down. Dates on the right side indicate last full day when CGM data were usable. Vehicle and liraglutide dosing days are indicated by dashed/long arrows pointing upward.

Supplementary Fig. S2. Mean and standard deviation of glucose levels over the first 5 weeks of CGM. Each symbol represents a single monkey. Diabetic monkeys are in blue (clear symbols, ID numbers beginning with 16) and healthy monkeys in orange (filled symbols, ID numbers beginning with 15). .

Supplementary Fig. S3. **(A)** Mean MSE curves and **(B)** mean MSE−$AUC_{10s-30m}$ for healthy and diabetic animals, using the first five weeks of CGM data.

Supplementary Fig. S4. MSE−$AUC_{10s-30m}$ of glucose by time of day , using the first five weeks of CGM data.

Supplementary Fig. S5. Within−animal variability of MSE curves for glucose. MSE curves calculated using data for **(A)** consecutive 3−day periods or **(B)** consecutive 1-week periods, during the first five weeks of CGM.

Supplementary Fig. S6. Within−animal variability of MSE−$AUC_{10s-30m}$ for glucose (Consecutive 3−day and 1−week periods during the first five weeks of CGM).

Supplementary Fig. S7. Within−animal SD of MSE−$AUC_{10s-30m.}$ Each symbol represents a single monkey. Diabetic monkeys are in blue (clear symbols, ID numbers beginning with 16) and healthy monkeys in red (filled symbols, ID numbers beginning with 15).

Supplementary Fig. S8. Liraglutide PK in rhesus. Liraglutide was injected subcutaneously at 200 µg/kg (N=3) and levels of liraglutide in blood determined.

Supplementary Tables

Supplementary Table S1. Overview of CGM data.

Supplementary Table S2. MSE of glucose for healthy and diabetic animals. Data are from the first five weeks of monitoring.

Supplementary Table S3. MSE− $AUC_{10s-30m}$ of glucose for healthy and diabetic animals. Data are from the first five weeks of monitoring.

Supplementary Table S4. Mean MSE−$AUC_{10s-30m}$ of glucose by time of day, for healthy and diabetic animals. Data are from the first five weeks of monitoring.

References

1 Costa, M., Goldberger, A. & Peng, C. K. *Phys. Rev. Lett.* **89**, 068102-068102 (2002).

2 Richman, J. S. & Moorman, J. R. Physiological time-series analysis using approximate entropy and sample entropy. *American journal of physiology. Heart and circulatory physiology* **278**, H2039-2049, doi:10.1152/ajpheart.2000.278.6.H2039 (2000).

3 Costa, M. D., Henriques, T., Munshi, M. N., Segal, A. R. & Goldberger, A. L. Dynamical glucometry: use of multiscale entropy analysis in diabetes. *Chaos* **24**, 033139 (2014).

4 Chen, J. L., Chen, P. F. & Wang, H. M. Decreased complexity of glucose dynamics in diabetes: evidence from multiscale entropy analysis of continuous glucose monitoring system data. *Am J Physiol Regul Integr Comp Physiol* **307**, R179-183 (2014).

5 Brockway, R. *et al.* Fully Implantable Arterial Blood Glucose Device for Metabolic Research Applications in Rats for Two Months. *J Diabetes Sci Technol* **9**, 771-781 (2015).

Supplementary Figure S1A: CGM timeline for four healthy rhesus monkeys

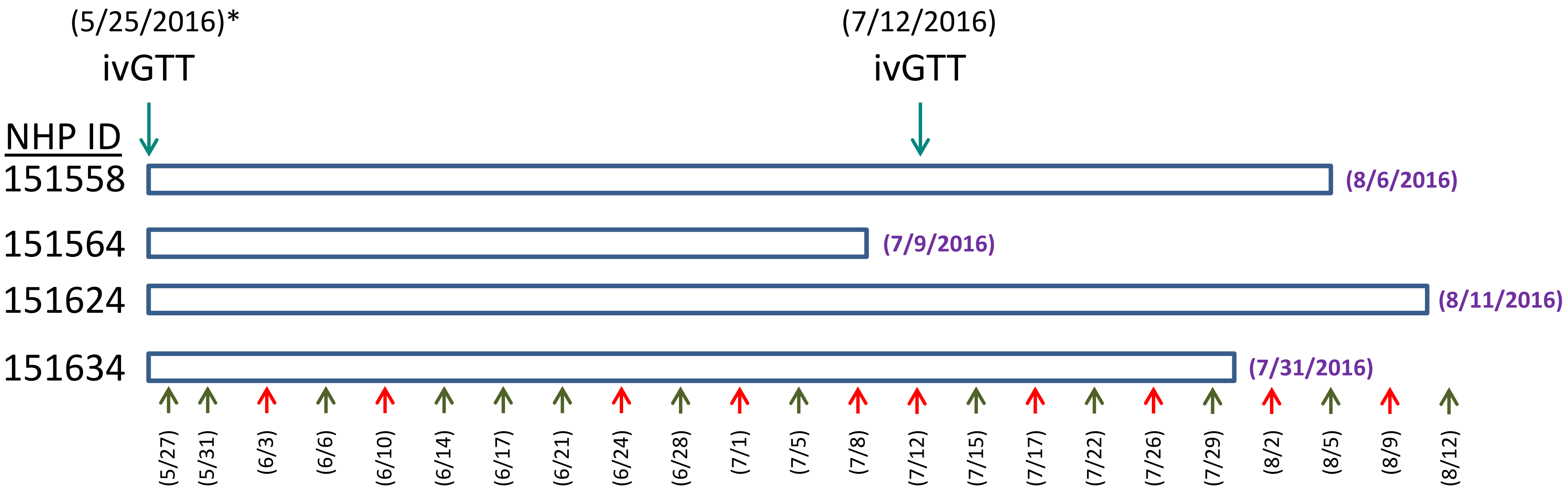

↑ (red): sedation/battery change/glucometer reading

Dates: Last whole day of usable data

↑ (green): glucometer reading

*: 5/27/2016 for NHP 151558

Supplementary Figure S1B: CGM timeline for six diabetic rhesus monkeys

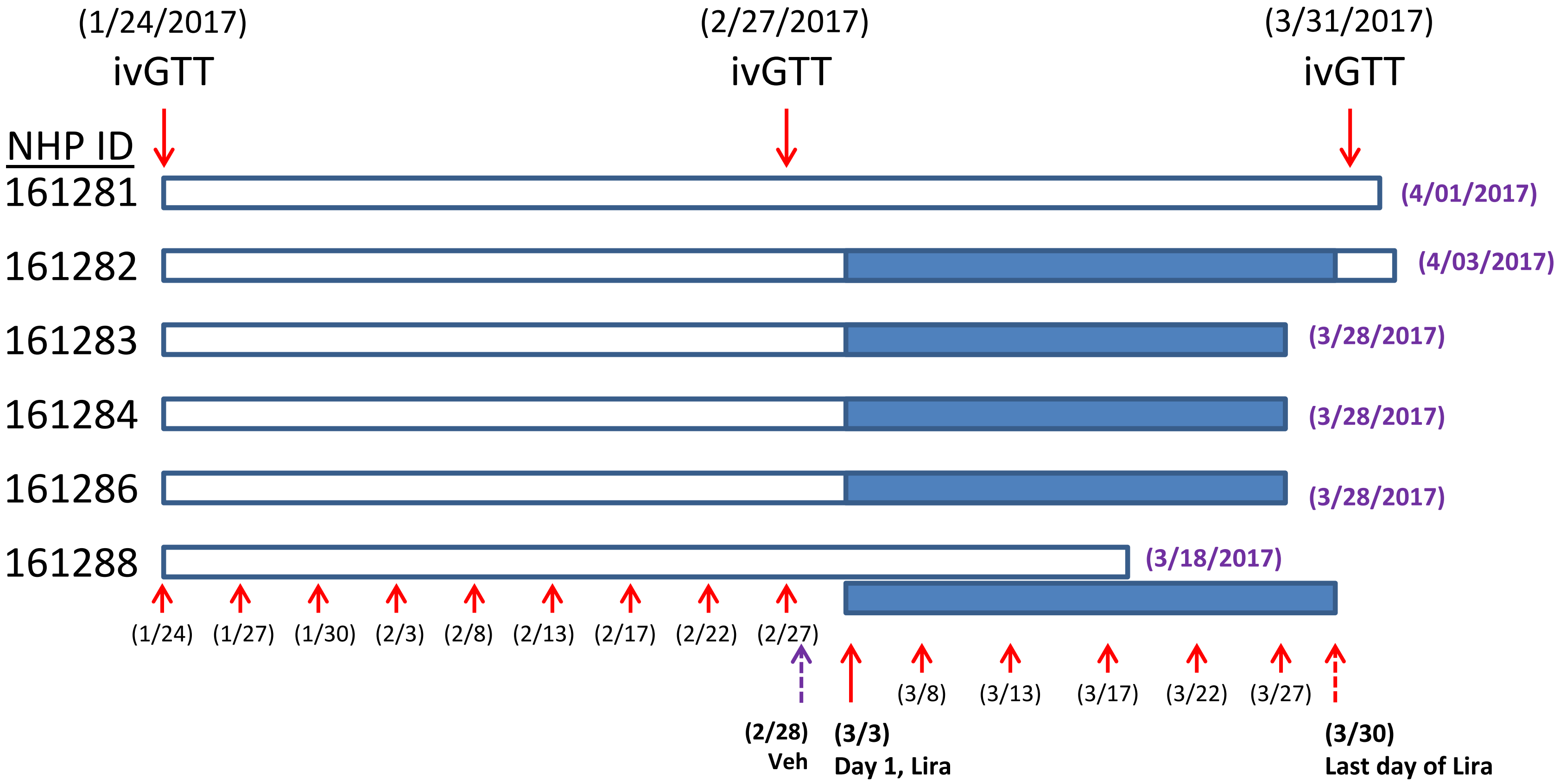

: Sedation/battery change/glucometer reading

: Lira dosing days

Date: Last whole day of usable data

Supplementary Figure S2. Mean and standard deviation of glucose over the first 5 weeks of monitoring

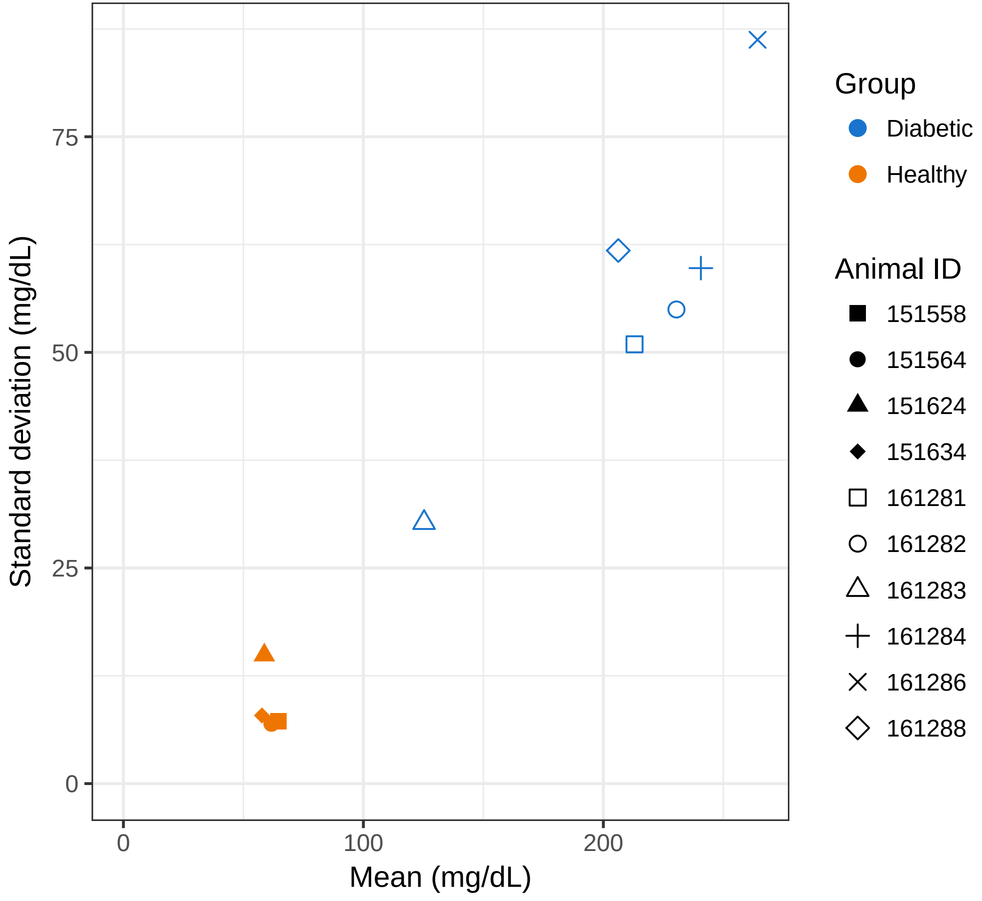

Supplementary Figure S3. Mean MSE curves and MSE−AUC for the first five weeks of monitoring

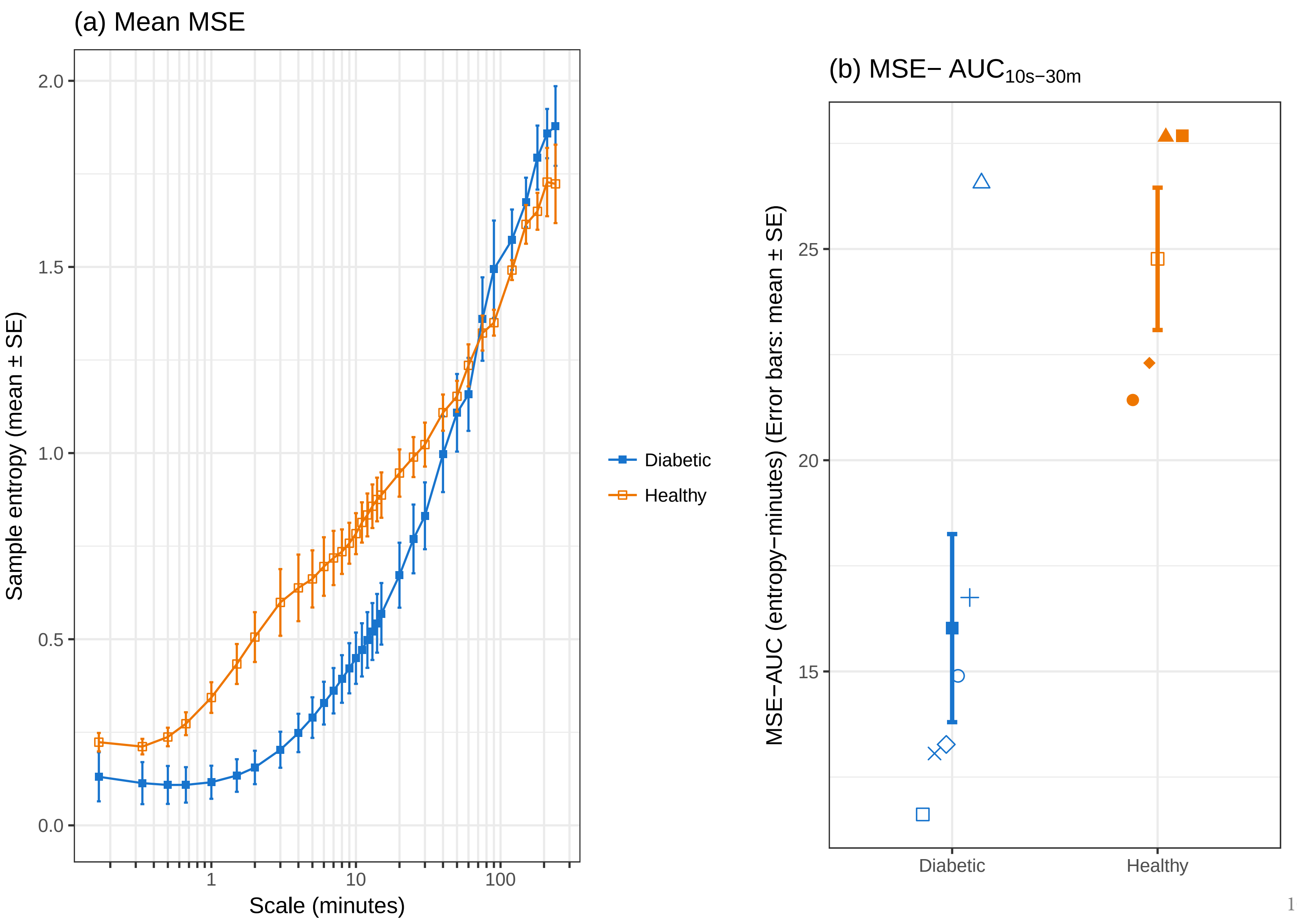

Supplementary Figure S4. MSE−AUC$_{10s-30m}$ of glucose, by time of day

Days 1−35 of monitoring

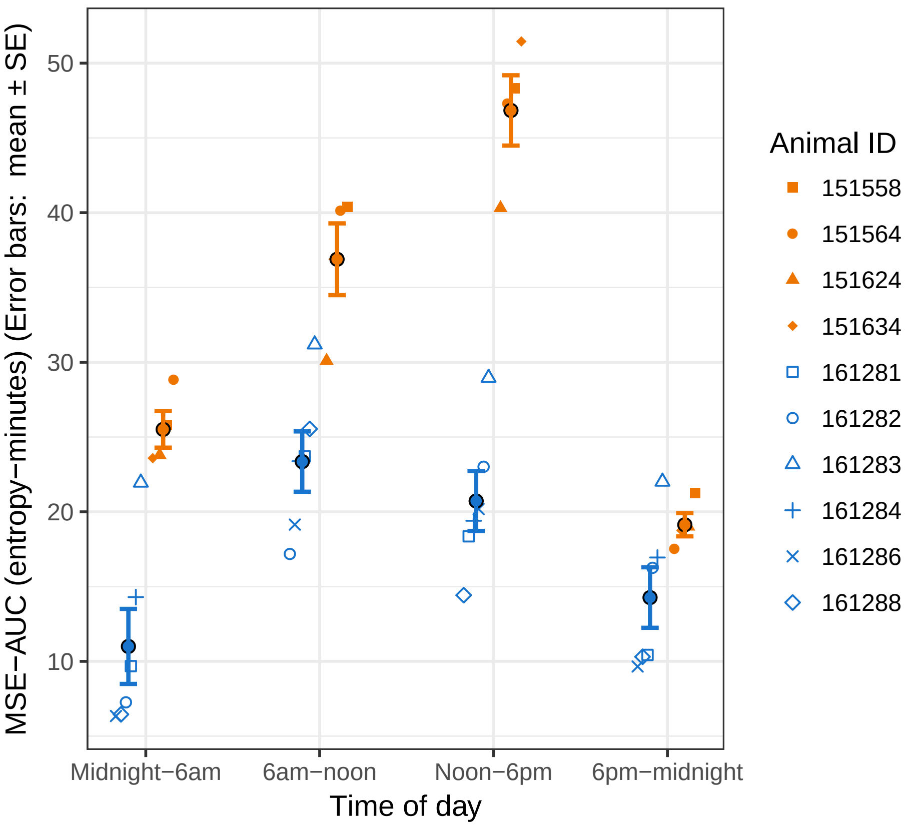

Supplementary Figure S5(a). Within−animal variability of MSE curves for glucose

Consecutive 3−day periods during the first five weeks of monitoring

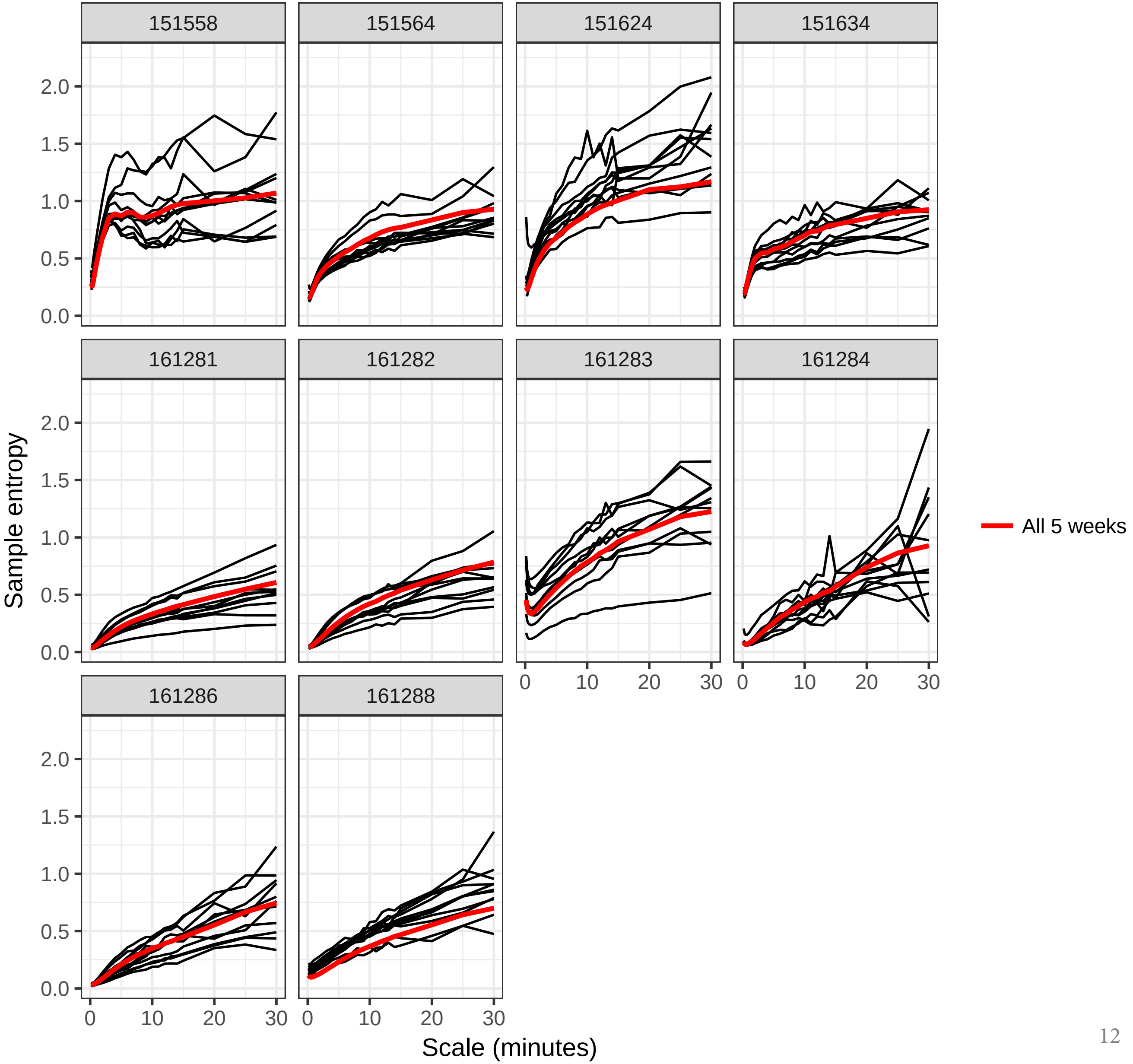

Supplementary Figure S5(b). Within−animal variability of MSE curves for glucose

Consecutive 1−week periods during the first five weeks of monitoring

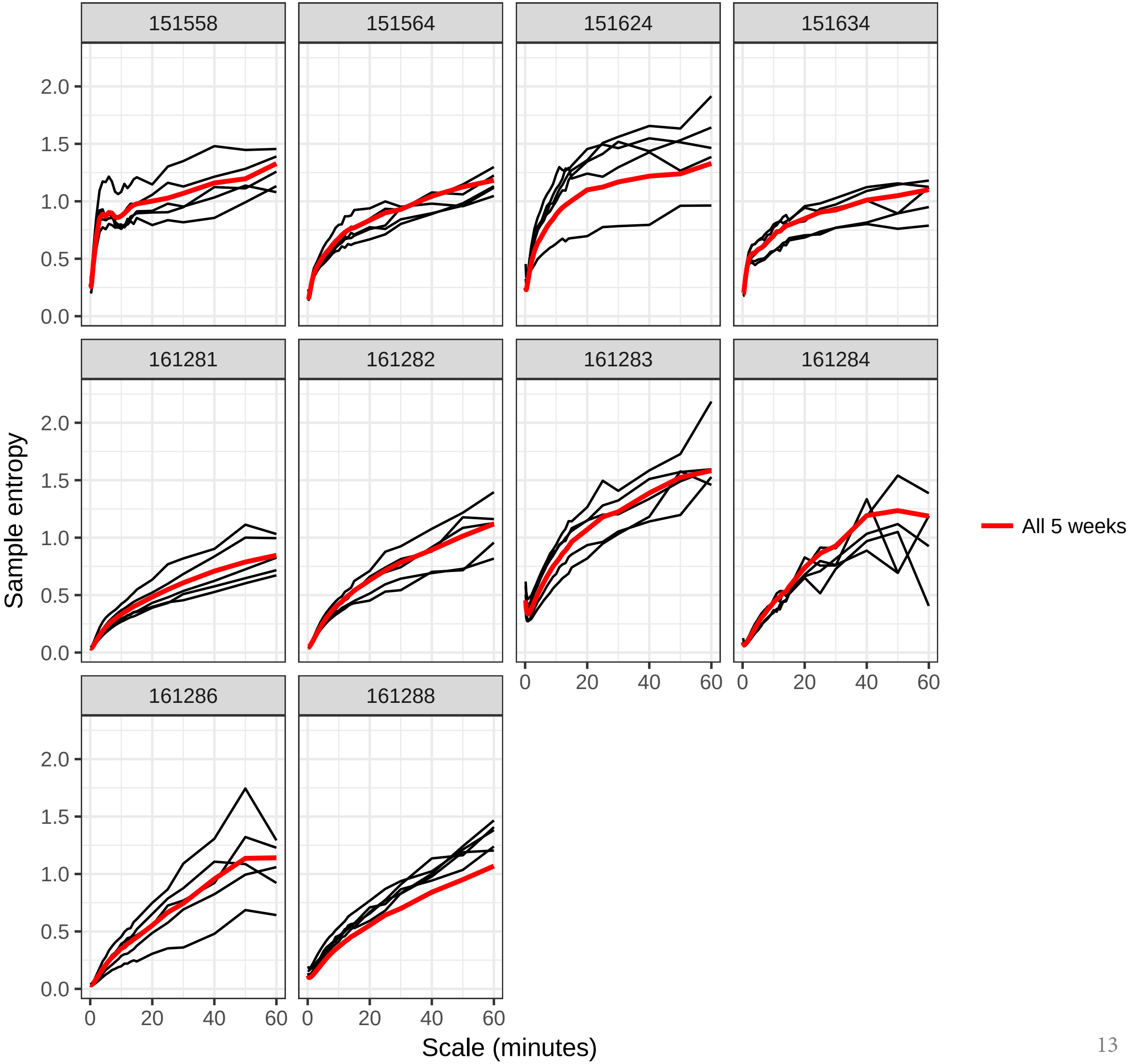

Supplementary Figure S6. Within−animal variability of MSE−AUC$_{10s-30m}$ for glucose

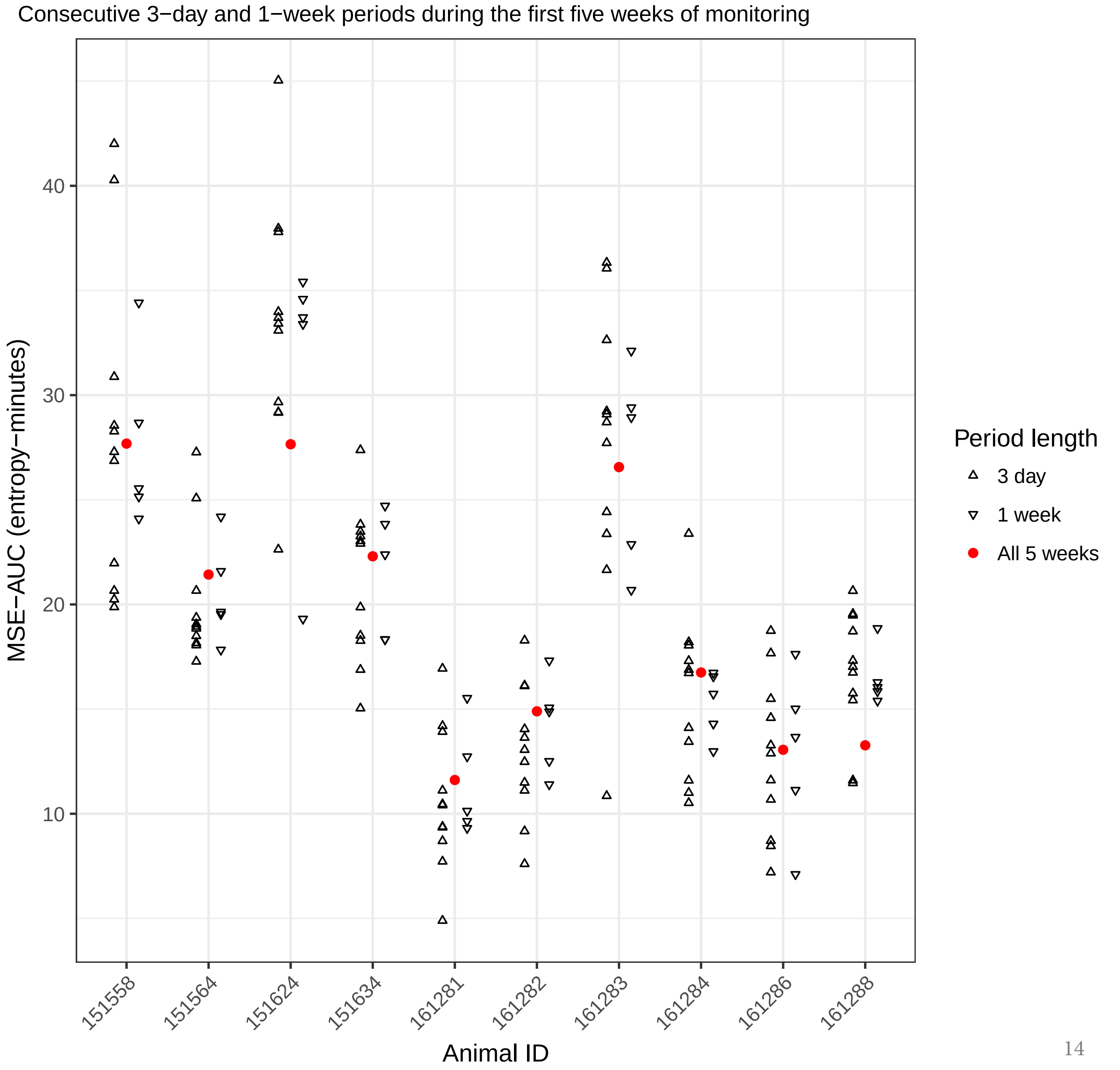

Supplementary Figure S7. Within−animal SD of MSE−AUC$_{10s-30m}$

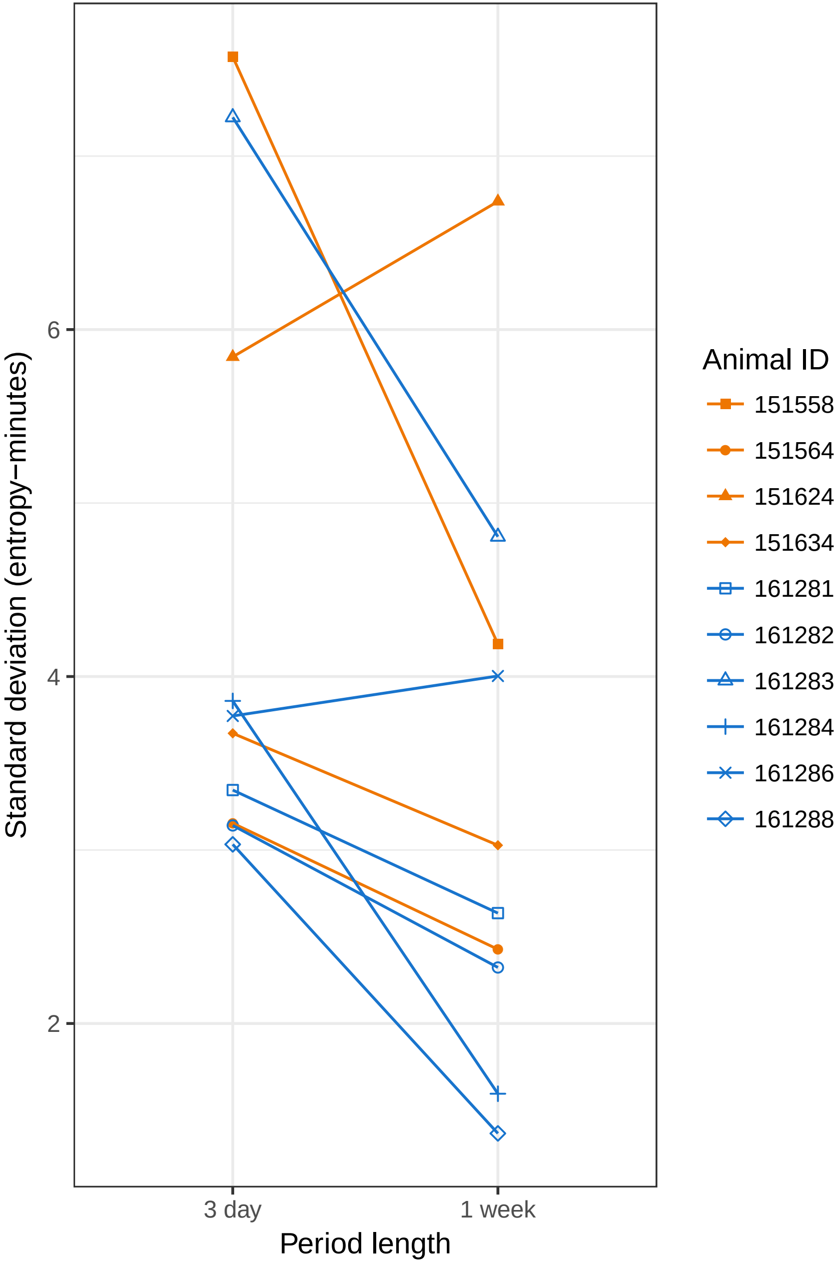

Supplementary Figure S8: liraglutide PK in rhesus following subcutaneous dosing

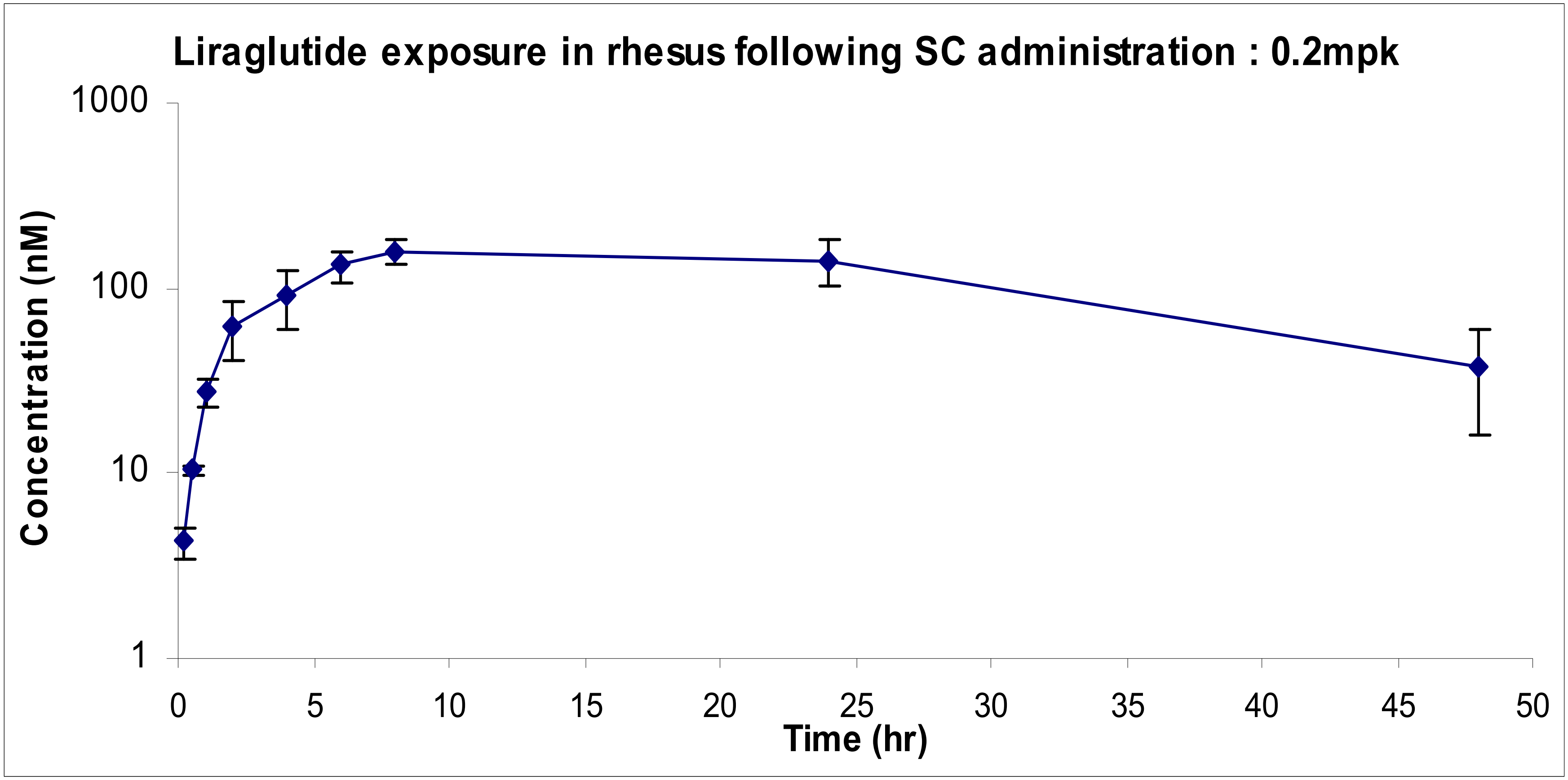

Species	Dose *(mg/kg)*	$AUC_{0-\infty}$ *(µM·h)*	C_{max} *(nM)*	T_{max} *(h)*	F *(%)*
Rhesus	0.2	5.7 ± 1.3	176 ± 5	13 ± 9	25

Supplementary Table S1. Overview of continuous glucose monitoring

Group	Animal ID	Sex	Age (years)	Weight (kg)	Usable CGM period (days)	Length of liraglutide treatment within usable CGM period (days)
Healthy	151558	M	4	5.6	73.5	N/A
	151564	M	4	6.8	45.5	N/A
	151624	M	4	6.0	78.5	N/A
	151634	M	4	6.0	67.6	N/A
Diabetic	161281	F	15	11.6	67.5	N/A
	161282	M	17	17.4	69.5	28.0
	161283	F	15	12.4	63.6	25.6
	161284	M	19	16.6	63.5	25.6
	161286	M	16	17.9	63.5	25.6
	161288	M	19	15.5	53.5	15.6

N/A: not applicable

Supplementary Table S2. MSE of glucose for healthy and diabetic animals

Data from the first five weeks of monitoring

	All animals									Excluding animal 161283							
	Diabetic				Healthy				Difference	Diabetic				Healthy				Difference
Scale (minutes)	N	Mean	SD	SEM	N	Mean	SD	SEM	P−value	N	Mean	SD	SEM	N	Mean	SD	SEM	P−value
0.17	6	0.13	0.16	0.07	4	0.22	0.05	0.02	0.233	5	0.07	0.04	0.02	4	0.22	0.05	0.02	0.003
0.33	6	0.11	0.14	0.06	4	0.21	0.04	0.02	0.152	5	0.06	0.03	0.01	4	0.21	0.04	0.02	0.001
0.50	6	0.11	0.12	0.05	4	0.24	0.05	0.02	0.057	5	0.06	0.03	0.01	4	0.24	0.05	0.02	0.002
0.67	6	0.11	0.12	0.05	4	0.27	0.06	0.03	0.021	5	0.06	0.02	0.01	4	0.27	0.06	0.03	0.004
1.00	6	0.12	0.11	0.04	4	0.34	0.08	0.04	0.006	5	0.07	0.02	0.01	4	0.34	0.08	0.04	0.006
1.50	6	0.13	0.11	0.04	4	0.43	0.11	0.05	0.004	5	0.09	0.02	0.01	4	0.43	0.11	0.05	0.007
2.00	6	0.16	0.11	0.04	4	0.51	0.13	0.07	0.006	5	0.11	0.02	0.01	4	0.51	0.13	0.07	0.009
3.00	6	0.20	0.12	0.05	4	0.60	0.18	0.09	0.013	5	0.16	0.02	0.01	4	0.60	0.18	0.09	0.015
4.00	6	0.25	0.13	0.05	4	0.64	0.18	0.09	0.013	5	0.20	0.02	0.01	4	0.64	0.18	0.09	0.016
5.00	6	0.29	0.13	0.05	4	0.66	0.15	0.08	0.008	5	0.24	0.02	0.01	4	0.66	0.15	0.08	0.011
6.00	6	0.33	0.14	0.06	4	0.70	0.16	0.08	0.009	5	0.27	0.03	0.01	4	0.70	0.16	0.08	0.011
7.00	6	0.36	0.15	0.06	4	0.72	0.15	0.07	0.008	5	0.30	0.03	0.01	4	0.72	0.15	0.07	0.009
8.00	6	0.39	0.16	0.06	4	0.74	0.12	0.06	0.005	5	0.33	0.04	0.02	4	0.74	0.12	0.06	0.005
9.00	6	0.42	0.16	0.07	4	0.76	0.11	0.05	0.005	5	0.36	0.04	0.02	4	0.76	0.11	0.05	0.003
10.00	6	0.45	0.17	0.07	4	0.78	0.11	0.06	0.005	5	0.38	0.05	0.02	4	0.78	0.11	0.06	0.003
11.00	6	0.47	0.18	0.07	4	0.81	0.11	0.05	0.005	5	0.40	0.05	0.02	4	0.81	0.11	0.05	0.002
12.00	6	0.50	0.18	0.07	4	0.83	0.12	0.06	0.007	5	0.43	0.05	0.02	4	0.83	0.12	0.06	0.003
13.00	6	0.52	0.19	0.08	4	0.86	0.12	0.06	0.008	5	0.45	0.06	0.03	4	0.86	0.12	0.06	0.003
14.00	6	0.54	0.19	0.08	4	0.88	0.12	0.06	0.010	5	0.47	0.06	0.02	4	0.88	0.12	0.06	0.003
15.00	6	0.57	0.20	0.08	4	0.89	0.12	0.06	0.015	5	0.49	0.07	0.03	4	0.89	0.12	0.06	0.003
20.00	6	0.67	0.21	0.09	4	0.95	0.13	0.06	0.035	5	0.59	0.10	0.04	4	0.95	0.13	0.06	0.004
25.00	6	0.77	0.23	0.09	4	0.99	0.11	0.05	0.076	5	0.69	0.12	0.05	4	0.99	0.11	0.05	0.005
30.00	6	0.83	0.22	0.09	4	1.02	0.12	0.06	0.114	5	0.75	0.12	0.05	4	1.02	0.12	0.06	0.012
40.00	6	1.00	0.25	0.10	4	1.11	0.10	0.05	0.355	5	0.92	0.18	0.08	4	1.11	0.10	0.05	0.085
50.00	6	1.11	0.26	0.10	4	1.15	0.08	0.04	0.706	5	1.02	0.17	0.08	4	1.15	0.08	0.04	0.195
60.00	6	1.16	0.24	0.10	4	1.24	0.11	0.06	0.512	5	1.07	0.13	0.06	4	1.24	0.11	0.06	0.088
75.00	6	1.36	0.27	0.11	4	1.32	0.09	0.05	0.766	5	1.27	0.20	0.09	4	1.32	0.09	0.05	0.643
90.00	6	1.49	0.32	0.13	4	1.35	0.07	0.03	0.331	5	1.43	0.32	0.14	4	1.35	0.07	0.03	0.592
120.00	6	1.57	0.20	0.08	4	1.49	0.05	0.03	0.376	5	1.54	0.20	0.09	4	1.49	0.05	0.03	0.651
150.00	6	1.67	0.16	0.07	4	1.61	0.10	0.05	0.502	5	1.68	0.18	0.08	4	1.61	0.10	0.05	0.546
180.00	6	1.79	0.21	0.09	4	1.65	0.10	0.05	0.187	5	1.78	0.23	0.10	4	1.65	0.10	0.05	0.302
210.00	5	1.86	0.15	0.07	4	1.73	0.18	0.09	0.295	4	1.83	0.16	0.08	4	1.73	0.18	0.09	0.415
240.00	5	1.88	0.24	0.11	4	1.72	0.21	0.11	0.335	4	1.90	0.27	0.14	4	1.72	0.21	0.11	0.357

P−values are from a 2−sided t−test comparing group means, not assuming equal variances for the two groups.
SD = standard deviation, SEM = standard error of mean

Supplementary Table S3. MSE− $AUC_{10s-30m}$ of glucose for healthy and diabetic animals

Data from the first five weeks of monitoring

	Diabetic				Healthy				Difference
	N	Mean	SD	SEM	N	Mean	SD	SEM	P−value
All animals	6	16.03	5.45	2.23	4	24.77	3.37	1.68	0.014
Excluding animal 161283	5	13.92	1.96	0.88	4	24.77	3.37	1.68	0.003

P−values are from a 2−sided t−test, not assuming equal variances for the two groups.
SD = standard deviation, SEM = standard error of mean, s = seconds, m = minutes

Supplementary Table S4. Mean MSE−$AUC_{10s-30m}$ of glucose by time of day, for healthy and diabetic animals

Days 1−35 of monitoring

	Diabetic				Healthy				Difference
Time of day	N	Mean	SD	SEM	N	Mean	SD	SEM	P−value
Midnight−6am	6	11.00	6.14	2.51	4	25.51	2.44	1.22	0.001
6am−noon	6	23.36	4.94	2.02	4	36.89	4.80	2.40	0.004
Noon−6pm	6	20.72	4.91	2.00	4	46.84	4.70	2.35	<0.001
6pm−midnight	6	14.27	4.96	2.02	4	19.13	1.55	0.77	0.063

P−values are from a 2−sided t−test, not assuming equal within−group variances.
SD = standard deviation, SEM = standard error of mean
